# Eating Disorders in School-Age Children During the COVID-19 Pandemic

**DOI:** 10.3390/children13020273

**Published:** 2026-02-16

**Authors:** Natasa Djorić, Ivan Vukosavljević, Ivana Vukosavljević, Igor Sekulić, Jelena Bošković Sekulić, Nebojša Zdravković, Neda Milosavljević, Šćepan Sinanović, Olivera Kostić

**Affiliations:** 1 Community Health Centre, 35000 Jagodina, Serbia; natasa.djoric@gmail.com (N.D.); ivanvukosavljevic2@gmail.com (I.V.); 2 Department in Ćuprija, Academy of Applied Preschool Teaching and Health Studies, 35230 Ćuprija, Serbia; ivana.vukosavljevic035@gmail.com; 3 Medical Faculty of the Military Medical Academy Belgrade, University of Defence, 11000 Belgrade, Serbia; igorsekulic76@gmail.com; 4 Institute of Radiology, Military Medical Academy, 11000 Belgrade, Serbia; 5 Clinic for Gastroenterology and Hepatology, University Clinical Center Kragujevac, 34000 Kragujevac, Serbia; jelenaboskovic76@gmail.com; 6 Department of Biomedical Statistics and Informatics, Faculty of Medical Sciences, University of Kragujevac, 34000 Kragujevac, Serbia; nzdravkovic@gmail.com; 7 Department of Clinical Oncology, Faculty of Medical Sciences, University of Kragujevac, 34000 Kragujevac, Serbia; neda.milosavljevic@yahoo.com; 8 High Medical College of Professional Studies “Milutin Milanković”, 11000 Belgrade, Serbia; scepan.sinanovic@gmail.com; 9 Department of Pharmacy, Faculty of Medical Sciences, University of Kragujevac, 34000 Kragujevac, Serbia

**Keywords:** COVID-19 pandemic, eating disorder, obesity, children, adolescents, EAT-26, DASS-21

## Abstract

(1) Background: Eating disorder risk factors in children are early maturation, body dissatisfaction, dieting, stress and physical inactivity. The COVID-19 pandemic has further exacerbated these factors due to isolation, online classes and reduced physical activity, all of which have increased children’s risk of developing eating disorders. The aim of the research was to examine the frequency of eating disorders among school-aged children in the Republic of Serbia during the COVID-19 pandemic, as well as the association of these disorders with socio-demographic characteristics, lifestyle habits, and levels of depression, anxiety and stress. (2) Methods: The research was conducted as a descriptive cross-sectional study on a sample of students from the fifth grade of elementary school to the fourth year of secondary school. The research was conducted from May to August in 2023. using the EAT-26 questionnaire. Before the research, the approval of the ethics committee of the Jagodina Health Center (No. 1238/28.04.2023.) was obtained, where the research was conducted. (3) Results: The results show that 5.8% of students exhibited eating disorder symptoms during the COVID-19 pandemic (EAT-26 ≥ 20). Statistically significant differences were observed in girls with an eating disorder, who had a significantly lower body weight compared to the others (*p* < 0.05). Students with symptoms of depression, anxiety and stress showed eating disorders significantly more often. Also, elementary school students and boys with an eating disorder visited a nutritionist and played sports more often. (4) Conclusions: Research has shown that during the COVID-19 pandemic, a smaller percentage of students showed symptoms of eating disorders, with girls being more sensitive. Disorders were significantly associated with the presence of depression, anxiety and stress. The obtained results indicate the importance of monitoring children’s psychological and nutritional health, as well as the need for preventive and intervention measures in crisis conditions.

## 1. Introduction

Appropriate eating habits should be adopted by children of preschool age. A balanced diet is necessary in order to preserve health and ensure the proper development of children. Food intake provides energy necessary for basal metabolism, as well as for all biochemical and metabolic processes in the body through the intake of proteins, fats, carbohydrates, vitamins, minerals and trace elements. Diseases of deficient nutrition appear due to the insufficient intake of certain nutritional elements in the case of improper nutrition, and on the other hand, due to the excessive intake of certain elements, diseases of sufficient nutrition appear [1]. Recent research shows that eating disorders among adolescents have increased in prevalence, particularly during and after the COVID-19 pandemic, with notable increases in binge eating and other disordered eating behaviors. For example, nearly half of adolescents surveyed during the pandemic reported moderate to severe binge-eating behaviors, associated with pandemic-related stress and psychosocial changes [2].

Adolescence is a vulnerable period for the onset of disordered eating, partly due to biological and psychosocial changes, body image concerns, and changing peer and cultural influences. Evidence suggests that girls are more often affected by eating disorders than boys, and that rates of disordered eating behaviors, including binge eating and restrictive eating, are elevated during this life stage. The prevalence of anorexia among girls ranges up to 5%, while the prevalence of binge eating disorder ranges from 3.5% to 10% of the general population [3]. Anorexia nervosa and bulimia nervosa are well-established eating disorders defined in major diagnostic manuals, and their diagnostic criteria have evolved over time. The DSM-5 (2013) recognizes these and other feeding and eating disorders (e.g., OSFED and UFED) based on current clinical understanding [4].

This study is conceptually grounded in the DSM-5 diagnostic framework, which provides the current standard classification for eating disorders. However, according to the DSM-5 classification, cases that do not meet full diagnostic criteria for anorexia nervosa or bulimia nervosa are classified under Other Specified Feeding or Eating Disorders (OSFED) and Unspecified Feeding or Eating Disorders (UFED). The OSFED category includes clinically significant presentations, such as atypical anorexia nervosa, subthreshold bulimia nervosa, purging disorder, and night eating syndrome, while binge eating disorder is recognized as an independent diagnosis in the DSM-5. In DSM-5, such cases are classified as OSFED or UFED, depending on whether specific diagnostic features are present but incomplete, or insufficient information is available to make a precise diagnosis [5]. This diagnosis also includes binge eating disorder, which is increasingly appearing as a separate eating disorder. The prevalence of binge eating disorder ranges from 0.7% to 4% in the general population [6].

Eating disorders are conditioned by the existence of biological, psychological and social factors, as well as economic and cultural influences, stressful events and dieting. Girls who mature earlier are more often dissatisfied with their bodies and resort to diets more often than girls who mature later. Due to the accumulation of fat deposits in certain body parts, girls at puberty are often more dissatisfied with their bodies than boys. Depending on the mentioned factors, an eating disorder can have a transient or chronic character [7,8]. The problem of children’s nutrition is becoming more and more relevant with the high availability of fast food, snacks based on sweets and carbonated drinks. In addition to improper nutrition, physical inactivity among children is increasingly pronounced due to spending too much time using mobile phones and computers. The biopsychosocial model posits that eating disorders arise from an interplay of biological, psychological, and socio-environmental factors, including stress, changes in eating habits, sedentary behaviors, and psychosocial pressures. During the COVID-19 pandemic, these stressors were intensified, and stress-related increases in binge-eating behaviors were observed among adolescents. This problem has become particularly pronounced with the emergence of the COVID-19 pandemic. During the pandemic, children often attended online classes. Also, children who were covid positive or someone from their family was positive, were forced to be in isolation, and they spent even more time on phones and computers, which together contribute to even greater physical inactivity. It is very important to solve the problem with children’s nutrition in the earliest period in order to ensure children’s proper growth and development.

Although international studies have examined the impact of the COVID-19 pandemic on adolescent eating behaviors, there is a lack of research specifically addressing the prevalence of eating disorders and their association with socio-demographic characteristics and psychological stress among school-aged children in Serbia. The aim of this research was to determine the frequency of eating disorders in school-aged children in primary and secondary schools in the territory of the Republic of Serbia during the COVID-19 pandemic. Also, the goal was to analyze the impact of socio-demographic characteristics and children’s habits, as well as depression, anxiety and stress, during the COVID-19 pandemic on eating disorders.

## 2. Materials and Methods

The research was carried out as a descriptive cross-sectional study of school-age children from May to August in 2023. The children were from the fifth to the eighth grade of primary school and from the first to the fourth year of secondary school in the city of Jagodina, selected during routine medical check-ups conducted at the Community Health Center Jagodina. Respondents were selected using a random sample method. The EAT-26 questionnaire was administered to teachers, who then distributed it to children who wished to participate in the research.

Before filling out the survey, how to fill out the survey was explained to the children, and it was emphasized that the survey is anonymous and that their answers will be used exclusively for the purposes of scientific research.

The criteria for participation of children in the study were that they were between the ages of 10 and 18, that is, that they attended the 5th to the 8th grade of elementary school, or that they were secondary school students. The exclusion criteria were that the children attended school according to the IEP, and that they could not independently fill out the survey with understanding.

The research was carried out after receiving approval for the research from the Ethics Committee of the Community Health Center Jagodina no. 1238, on 28 April 2023. As the study participants included children (participants of school age), in accordance with ethical principles, consent was obtained from their parents or legal guardians for participation in the survey. Ethical research standards are in line with international (Declaration of Helsinki) and the specific legislation of the Republic of Serbia. In order to respect the privacy of the research subject and the confidentiality of information, all necessary steps were taken in accordance with the Law on the Protection of Personal Data, the Law on Official Statistics and the Directive of the European Parliament on the Protection of Personal Data (Directive 95/46/EC).

The measurement instrument used during the research was a questionnaire consisting of three parts. The first part of the questionnaire is the Eating Attitudes Test—EAT-26 questionnaire [9]. It includes the socio-demographic characteristics of children and their families, their satisfaction with their appearance, and their physical activity.

The EAT-26 questionnaire assesses respondents’ satisfaction with body appearance and weight and their attitudes and behaviors related to nutrition and their body. The EAT-26 questionnaire was used with the author’s permission for this purpose. Nevertheless, the EAT-26 questionnaire has been validated in the Croatian language [10], and that version of the questionnaire was adapted to the Serbian language and used in this study due to the similarity in language and territorial characteristics of participants. This questionnaire provides us with efficient information for research purposes, but it is not used for diagnosis. The questionnaire consists of 26 items, and the total EAT-26 score is calculated as the sum of the individual items and ranges from 0 to 78. The answers are defined using a six-point Likert scale, with answers ranging from always to never. The answer “always” receives 3 points, the answer “very often” receives 2 points, the answer “often” receives 1 point, and the answers “rarely”, “sometimes” and “never” receive 0 points. A score greater than or equal to 20 indicates an eating disorder. The EAT-26 questionnaire consists of three subscales related to dieting, bulimia and oral food control, which provides us with additional information about eating disorders.

The second part of the questionnaire refers to children’s eating habits and physical activity during the COVID-19 pandemic.

The third part of the questionnaire is the DASS-21 scale (Depression Anxiety Stress Scales). This is a standardized self-report scale for symptoms of depression, anxiety and stress. It consists of a set of 3 subscales with 7 questions each, designed to assess states of depression, anxiety and stress. Respondents rated on a 4-point Likert-type scale how they felt in the last week, that is, the severity/frequency of symptoms of depression, anxiety and stress they had, from 0 (“not at all”) to 3 (“mostly or almost always”). Depression, anxiety and stress scores were obtained by summing the scores of the relevant items in the range of 0–21 for each subscale. Symptom severity was ranked using cut-off scores to define normal, mild, moderate, significant, and very significant scores for each subscale. A normal score (absence of symptoms) for the depression subscale is 0–4, anxiety 0–3, and stress 0–7. Mild symptomatology means a score for the depression subscale of 5–6, anxiety of 4–5, and stress of 8–9. Moderate symptomatology is defined by scores of 7–10 for the depression subscale, 6–7 for the anxiety subscale, and 10–12 for the stress subscale. Severe symptomatology is characterized by scores of 11–13 for the depression subscale, 8–9 for the anxiety subscale, and 13–16 for the stress subscale. Very serious symptomatology is defined by a score for the depression subscale of 14+, anxiety of 10+ and stress of 17+. The mentioned scores imply the degree of severity of symptoms and not the degree of mental disorder. The scale is in the public domain and its use does not require the author’s consent.

Dependent variables are the results of the EAT-26 questionnaire related to eating disorders in children, as well as the results of the DASS-21 scale related to depression, anxiety and stress in children during the COVID-19 pandemic.

Independent variables are those related to the child’s age, gender, socio-demographic characteristics, as well as other characteristics related to nutrition and physical activity, with special reference to the period during the COVID-19 pandemic.

Statistical processing of the data was performed using IBM SPSS Statistics version 23. For descriptive statistical analysis of continuous variables, the average value and standard deviation were used, and for categorical variables, absolute and relative frequency were used. The Kolmogorov–Smirnov normality test was used to check the normality of the data distribution. For the analysis of students’ eating disorders based on the results of the EAT-26 questionnaire in relation to continuous variables, the Student’s *t*-test for independent samples was used if the data followed a normal distribution and the Mann–Whitney U test if the data did not follow a normal distribution. A box plot was used to display the results graphically. A chi-square test for independence was used to analyze students’ eating disorders based on the results of the EAT-26 questionnaire in relation to categorical variables. A bar cluster chart was used for the graphical presentation of the results. The results were considered statistically significant if the r value was less than 0.05.

The study sample size was calculated using the statistical software G*Power 3.1 for a two-tailed Student’s t-test for independent samples, with an accepted Type I error probability of α = 0.05 and a statistical power of 0.95. Assuming equal group sizes, the total sample size was estimated at a minimum of 180 subjects. The sample size was calculated based on the data of a study of a similar design [11], starting from the value of the EAT-26 score, which was on average 15.3 ± 10.9 for girls and 10.7 ± 8.6 for boys.

The sample consisted of 189 school-age children attending primary and secondary schools in the Republic of Serbia. The sample consisted of 55% girls and 45% boys, as well as 36.5% of elementary school students and 63.5% of secondary school students. Based on the EAT-26 score, we divided the students into two groups: those who do not have an eating disorder with a score of up to 19 and those who have an eating disorder with a score of 20 and above.

## 3. Results

The average age of the students was 14.34 ± 2.64 years. Average body weight was 56.98 ± 12.32 kg, and height was 162.68 ± 14.83 cm. The body mass index (BMI) value was calculated on the basis of the student’s weight and height, with an average BMI value of 21.21 ± 3.85. Detailed values related to the socio-demographic characteristics of students are shown in Table 1.

Detailed distributions of BMI percentiles and nutritional status categories among study participants are presented in Table 2. BMI percentiles were calculated according to age- and sex-specific reference values. Nutritional status categories were defined using standard BMI percentile cut-offs.

During the COVID-19 pandemic, most children (60.3%) did not engage in any physical activity and slightly more than half of students (51.9%) ate at school, while most children consumed fast food (83.6%) and soft drinks (83.1%). For the majority of students (74.1%), eating habits during the pandemic did not differ from usual. About half of the students (50.3%) said that they gained weight during the pandemic, while the majority of children (94.2%) did not visit a nutritionist. An overview of students’ habits during the COVID-19 pandemic is shown in Table 3.

The score for the EAT-26 questionnaire ranged from 0 to 43, with the average value of the score being 7.36, which indicates that children generally did not have a problem with nutrition during the COVID-19 pandemic. Our analysis found that 94.2% of students did not have a problem with nutrition.

Through descriptive statistical analysis, we obtained a total score for depression that ranged from 0 to 20, with the average value of the score being 2.42; the total score for anxiety ranged from 0 to 16, with the average value being 2.89; while the total score for stress ranged from 0 to 21, with the average value being 5.04. Given that the average score values are quite low, we divided the students into two groups: the first consisted of students who had a complete absence of symptoms, and the second consisted of students who had some symptomatology. The results of this analysis are shown in Table 4.

The analysis was conducted separately for all students, and then separately by gender and by the school the students attend. The results of this research are shown in Table 5.

Using the Mann–Whitney U test, it was determined that there is a statistically significant difference in the weight of girls who have and do not have an eating disorder. Girls who have an eating disorder have a significantly lower body weight (M = 44.5 kg) compared to girls who do not have an eating disorder (M = 58 kg). The analysis was conducted separately for all students, and then separately by gender and separately in relation to the school the students attend. The results of this research are shown in Table 6.

**Figure 1:**
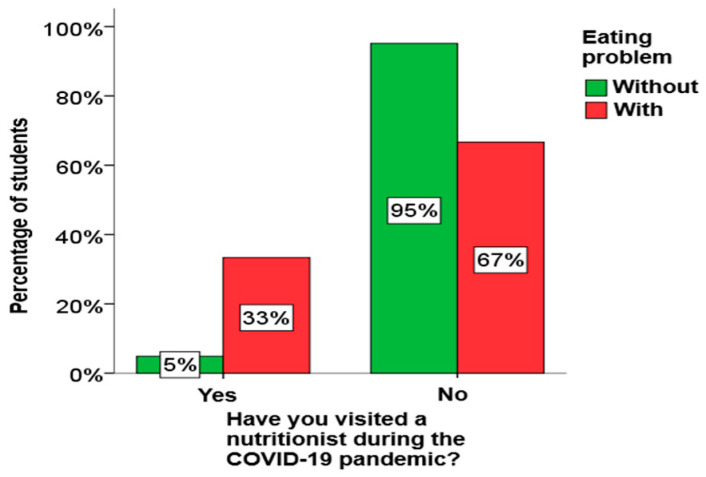
The impact of a visit to a nutritionist on eating disorders in boys.

**Figure 2:**
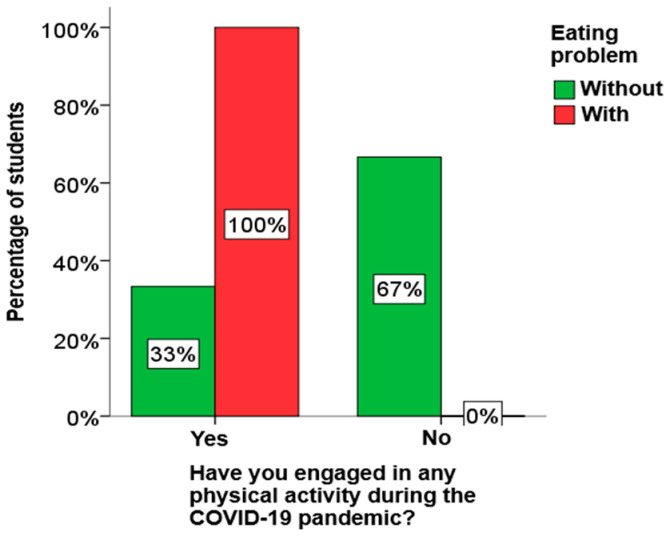
The influence of physical activity on eating disorders in primary school students.

**Figure 3:**
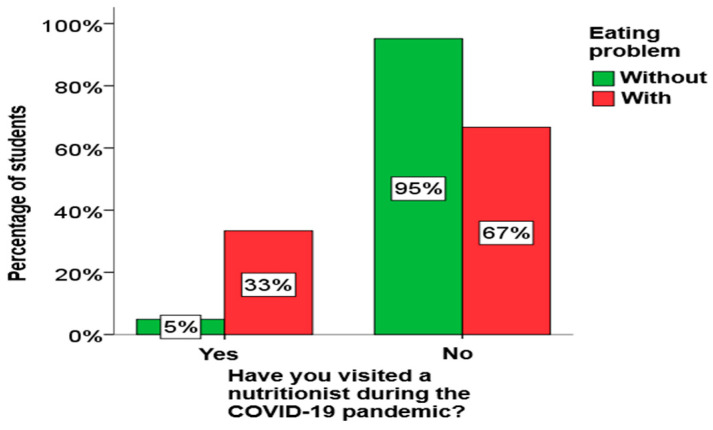
The influence of physical activity on eating disorders in secondary school students.

Using the χ2 test for independence, it was determined that there is a statistically significant relationship between eating disorders and nutritionist visits among boys, as well as between eating and physical activity problems and nutritionist visits among elementary school students. A graphical presentation of these results is given in Figure 1, Figure 2 and Figure 3.

From Figure 1, Figure 2 and Figure 3, it can be clearly seen that both boys and elementary school students who have an eating disorder visited a nutritionist much more often, as well as that all elementary school students who have an eating disorder play sports.

The chi-square test for independence was used to analyze eating disorders in students in relation to depression, anxiety and stress during the COVID-19 pandemic. The analysis was performed separately for all students, and then separately by gender and by the school the students attend. The results of this research are shown in Table 7.

**Figure 4:**
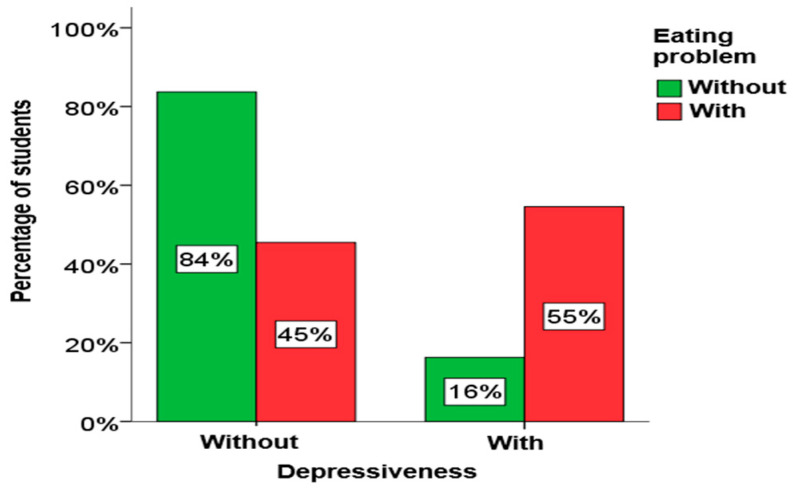
The influence of depression on the problems.

**Figure 5:**
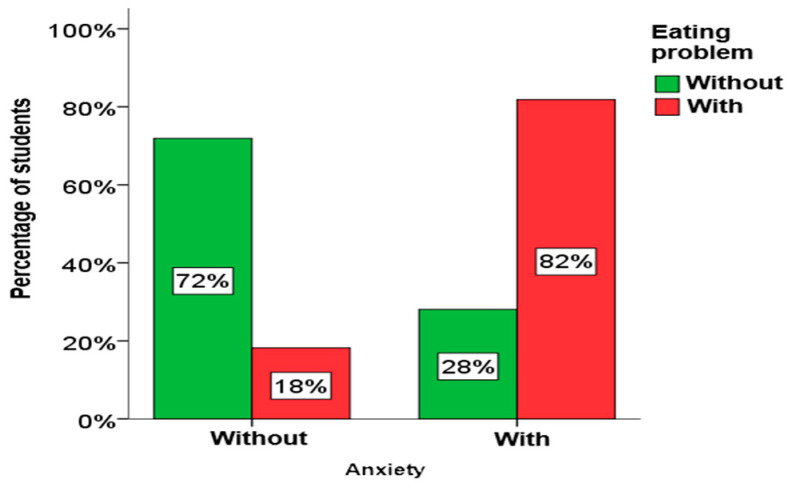
The influence of anxiety on eating disorders.

**Figure 6:**
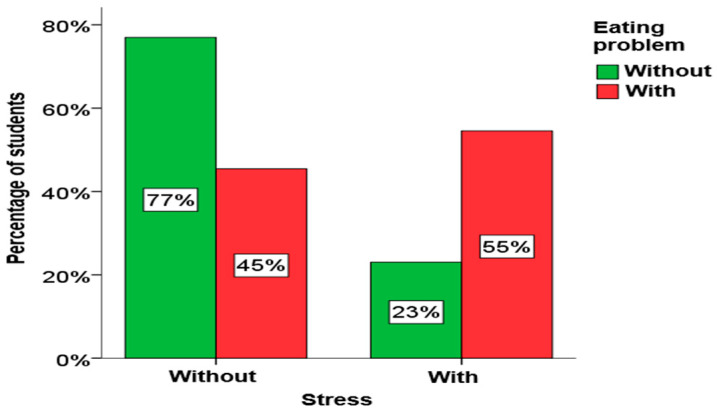
The influence of stress on eating disorders.

Using the chi-square test for independence, it was determined that there is a statistically significant relationship between depression, anxiety and stress in students and eating disorders. A significant connection between eating disorders and depression exists in both girls and boys, as well as among elementary school students. A significant connection between eating disorders and anxiety also exists in girls, as well as in elementary and secondary school students. A significant link between eating disorders and stress exists only in girls. A graphical presentation of these results is given in Figure 4, Figure 5 and Figure 6.

From Figure 4, Figure 5 and Figure 6, it is clearly seen that among students who have symptoms of depression, anxiety and stress, the percentage of students who have eating disorders is significantly higher.

To determine which variables had the greatest impact on eating disorders among all respondents, as well as separately for girls, boys, elementary, and secondary school students, binary logistic regression was applied. The results of this analysis are shown in Table 8.

The results indicate that depression had the largest estimated effect on eating disorders for all respondents, as well as for boys and secondary school students; however, these effects were not statistically significant (see Table 8, odds ratios and 95% confidence intervals). Among elementary school students, anxiety showed the largest estimated effect, but this effect was also not statistically significant. The only statistically significant predictor was body weight in girls, which had a meaningful effect on the likelihood of eating disorders (odds ratio = 0.90, 95% CI: 0.82–0.98, *p* < 0.05). The logistic regression model for girls showed a moderate ability to explain the variance in eating disorders, with Cox & Snell R^2^ = 0.151 and Nagelkerke R^2^ = 0.361. These values indicate that the model accounts for approximately 15% to 36% of the variability in the outcome.

## 4. Discussion

The results of our research indicate the presence of eating disorders in 5.8% of students during the COVID-19 pandemic, which is in line with the findings of previous research. A systematic review by Nasong et al. [12], published in the journal *Children and Youth Services Review*, indicates that as many as 83% of studies reported a negative impact of the pandemic on the nutrition of children and adolescents. Although the frequency in our sample appears lower than that reported in some other countries, such as Canada and the USA, where hospitalizations due to eating disorders increased during the pandemic [13,14], these findings should be interpreted cautiously, given the limited sample size and single-city scope of our study. They may indicate a trend, but generalization to the wider Serbian population is not warranted. A particularly significant finding is that girls with an eating disorder had a statistically significantly lower body weight (M = 44.5 kg) compared to girls without the disorder (M = 58 kg). This data is in line with research that indicates the increased vulnerability of girls during adolescence, especially in crisis situations such as a pandemic, when restrictive eating patterns and body dissatisfaction increase [15,16].

Furthermore, it was determined that students who exhibit symptoms of depression, anxiety and stress are significantly more likely to have an eating disorder. This is consistent with the findings of numerous studies that have shown that mental health is a key risk factor for the development of eating disorders. For example, a study published in the *Journal of Eating Disorders* showed that the pandemic led to an increase in the number of hospitalizations of adolescents, especially girls, due to eating disorders, with psychological symptoms playing a key role in the onset of the disorder [17]. Similarly, Racine et al. [18] and Slater et al. [19] indicate a high association between symptoms of anxiety and depression and the occurrence of restrictive and binge eating disorders.

One of the more interesting findings of our research refers to boys and elementary school students who have an eating disorder—they visit a nutritionist significantly more often, and all elementary school students from this group play sports. This information may indicate a greater awareness or concern for health in this population, but it may also point to hidden forms of disorders that are manifested through excessive concern about nutrition and physical appearance. Previous research indicates that eating disorders in boys often go unrecognized because they are motivated by muscle mass and body composition control, not necessarily weight loss [20,21]. Physical activity in adolescents with eating disorders, although apparently positive, can sometimes represent a form of compensation and weight control, especially in combination with dietary restriction.

Logistic regression analysis suggested that depression had the largest estimated effect on eating disorders in the overall sample (based on the Wald statistics), as well as in boys and secondary school students, while anxiety had the largest estimated effect in primary school students. However, none of these effects were statistically significant, indicating that these relationships should be interpreted with caution and may reflect trends rather than definitive associations. The only statistically significant predictor was body weight in girls, highlighting the relevance of weight-related factors in this subgroup. The only statistically significant predictor in the model was body weight in girls, which confirms the findings that low body weight is often directly related to eating disorders, especially in the female population. These results are in line with studies emphasizing the multifactorial nature of eating disorders, with psychological symptoms acting as risk factors, but often interacting with other psychosocial and physical factors [14,22].

Although the majority of students in our sample did not exhibit an eating disorder, the presence of risk factors, such as depressive and anxiety symptoms, suggests potential vulnerability. Given the study’s limited sample and geographic focus, these observations should be considered exploratory, highlighting areas for future research rather than conclusive trends in the post-pandemic population. Our findings confirm the need for early identification of risk groups, especially girls, students with symptoms of depression and anxiety, but also boys and younger students whose disorders are often more difficult to detect.

In this regard, the recommendations arising from this research refer to the need for systematic monitoring of mental health and eating habits in the school environment, as well as the development of preventive programs that include physical and psychological aspects of student well-being. An integrated approach that includes the cooperation of teachers, professional associates, parents and health workers can play a key role in the prevention and early detection of eating disorders among children and young people.

The main limitation of this study is its restriction to a single city and a relatively small sample, which limits the generalizability of the findings. Future studies should include a larger and more geographically diverse sample to verify whether the observed patterns hold across different regions of Serbia. In the context of this research, the limitation was justified due to the duration of the COVID-19 pandemic. An additional limitation was the use of the EAT-26 questionnaire using a similar language. Although the questionnaire has been previously used in similar regional studies, a full psychometric validation in the Serbian population has not yet been performed, which may affect the interpretation and generalizability of the results. Future research should include a formal validation study of the Serbian version of the EAT-26 to ensure reliability and construct validity in this population. At the end of the limitations section, we must also mention the cross-sectional design and self-reported data of our study design.

## 5. Conclusions

The results of this research indicate that during the COVID-19 pandemic, there was a moderate frequency of eating disorders in school-age children in the Republic of Serbia, with 5.8% of respondents showing symptoms of eating disorders according to the EAT-26 scale. Although the majority of students did not show symptoms, the data show that the pandemic affected the lifestyle, eating habits and mental state of children, which in some cases was reflected in the appearance of symptoms of eating disorders. It is particularly significant that the eating disorder was more common in girls, with the accompanying lower body weight, which confirms the already existing findings about the greater vulnerability of girls in the adolescent period.

Also, symptoms of depression, anxiety and stress showed a statistically significant association with eating disorders, which emphasizes the need for an integrated approach to mental health and nutrition when working with children and young people. It is surprising that boys and elementary school students who had an eating disorder were more often physically active and visited a nutritionist, which may indicate a different manifestation and experience of the disorder in these groups.

In all respondents, depression had the greatest unique influence on eating disorders, while in boys and secondary school students, the influence of depression was also observed, but without statistical significance. In elementary school students, anxiety had the greatest impact, but also without statistical significance. The only statistically significant predictor was body weight in girls, which further confirms its importance in the context of eating disorders.

Taking into account the negative effects of the pandemic on children’s lifestyles, this paper points out the importance of monitoring children’s nutritional behavior and psychological symptoms, especially in times of crisis. Preventive measures, such as education of students, parents and teachers, early identification of risky behaviors and adequate professional support, are of crucial importance for preserving the health of children and adolescents.

Further research should focus on the long-term effects of pandemics and similar crises on the mental and physical health of young people, with special reference to the development of healthy lifestyles, mechanisms of resilience and different patterns of manifestation of eating disorders in different demographic groups.

## Figures and Tables

**Table 1:** Socio-demographic characteristics of students.

	All Participants (N = 189)	Gilrs (N = 104)	Boys (N = 85)	Primary School Students (N = 69)	Secondary School Students (N = 120)
Age	M = 14.34 SD = 2.64	M = 14.83 SD = 1.97	M = 13.74 SD = 3.18	M = 11.54 SD = 2.38	M = 15.95 SD = 0.76
Weight	M = 56.98 SD = 15.32	M = 56.47 SD = 11.29	M = 57.60 SD = 19.19	M = 48.57 SD = 15.28	M = 61.82 SD = 13.14
Height	M = 162.68 SD = 14.83	M = 163.52 SD = 10.08	M = 161.65 SD = 19.12	M = 152.12 SD = 17.67	M = 168.76 SD = 8.18
Difference to the desired weight	M = 0.75 SD = 6.56	M = 1.56 SD = 6.10	M = −0.24 SD = 7.00	M = −0.20 SD = 4.82	M = 1.30 SD = 7.35
Number of sister and brothers at family	M = 1.41 SD = 1.48	M = 1.34 SD = 1.39	M = 1.51 SD = 1.59	M = 1.91 SD = 2.05	M = 1.13 SD = 0.92

M—Mean; SD—Standard deviation.

**Table 2:** BMI percentiles and nutritional status categories among study participants.

Group	*n*	BMI, 5th Percentile	BMI, 85th Percentile	Underweight *n* (%)	Normal Weight *n* (%)	Overweight *n* (%)
All participants	189	15.41	24.91	9 (4.76)	152 (80.42)	28 (14.81)
Girls	104	15.31	25.00	5 (4.81)	84 (80.77)	15 (14.42)
Boys	85	15.44	24.88	4 (4.71)	69 (81.18)	12 (14.12)
Primary school students	69	13.92	24.38	2 (2.90)	61 (88.41)	6 (8.70)
Secondary school students	120	16.82	25.31	1 (0.83)	109 (90.83)	10 (8.33)

BMI—body mass index.

**Table 3:** Students’ habits during the COVID-19 pandemic.

Questions	YES	NO
Have you engaged in any physical activity during the COVID-19 pandemic?	75 39.7%	114 60.3%
Have you been eating at school during the COVID-19 pandemic?	98 51.9%	91 48.1%
Have you consumed fast food during the COVID-19 pandemic?	158 83.6%	31 16.4%
Have you been drinking soft drinks during the COVID-19 pandemic?	157 83.1%	32 16.9%
Did your diet during the COVID-19 pandemic differ from your usual diet?	49 25.9%	140 74.1%
Have you gained weight during the COVID-19 pandemic?	95 50.3%	94 49.7%
Have you visited a nutritionist during the COVID-19 pandemic?	11 5.8%	178 94.2%

**Table 4:** Descriptive indicators of depression, anxiety and stress.

	Absence of Symptoms	Mild Symptomatology	Moderate Symptomatology	Serious Symptomatology	Very Serious Symptomatology
Depressiveness	81.48%	7.94%	7.94%	1.59%	1.06%
Anxiety	68.78%	12.17%	9.52%	3.17%	6.35%
Stress	75.13%	10.05%	6.35%	6.35%	2.12%

**Table 5:** Analysis of eating disorders in relation to socio-demographic characteristics of students.

In Relation to the EAT-26 Score	All Respondents (N = 189)	Girls (N = 104)	Boys (N = 85)	Primary School Students (N = 69)	Secondary School Students (N = 120)
Gender	1.149 0.224	-	-	0.519 0.471	0.804 0.370
School the student attends	0.430 0.512	0.135 0.713	0.132 0.717	-	-
How successful are you in school?	1.504 0.471	0.763 0.683	1.080 0.583	2.016 0.365	2.170 0.338
Are your parents married?	0.510 0.475	0.193 0.660	0.561 0.454	0.595 0.441	0.148 0.700
Do you do any physical activity?	0.013 0.911	0.207 0.649	1.878 0.171	3.845 0.050	1.403 0.236

The first value is the χ2 statistic; the second value is the significance (r).

**Table 6:** Analysis of eating disorders in relation to student habits during the COVID-19 pandemic.

In Relation to the EAT-26 Score	All Respondents (N = 189)	Girls (N = 104)	Boys (N = 85)	Primary School Students (N = 69)	Secondary School Students (N = 120)
Have you engaged in any physical activity during the COVID-19 pandemic?	1.078 0.299	0.406 0.524	0.921 0.337	5.520 0.019	0.061 0.805
Have you been eating at school during the COVID-19 pandemic?	0.034 0.854	0.013 0.910	0.277 0.599	0.264 0.607	0.010 0.922
Have you consumed fast food during the COVID-19 pandemic?	0.027 0.870	0.475 0.491	0.613 0.434	0.411 0.521	0.188 0.664
Have you been drinking soft drinks during the COVID-19 pandemic?	0.510 0.475	0.023 0.872	0.777 0.378	0.898 0.343	1.924 0.165
Did your diet during the COVID-19 pandemic differ from your usual diet?	0.011 0.916	0.193 0.860	1.340 0.247	0.003 0.956	0.031 0.861
Have you gained weight during the COVID-19 pandemic?	0.835 0.361	0.003 0.955	2.898 0.089	1.122 0.289	0.088 0.767
Have you visited a nutritionist during the COVID-19 pandemic?	0.228 0.633	0.531 0.466	4.233 0.040	4.355 0.037	0.631 0.466

The first value is the χ2 statistic; the second value is the significance (r).

**Table 7:** Analysis of eating disorders in relation to depression, anxiety and stress in students during the COVID-19 pandemic.

	All Respondents (N = 189)	Girls (N = 104)	Boys (N = 85)	Primary School Students (N = 69)	Secondary School Students (N = 120)
Depressiveness	10.047 0.002	5.844 0.016	4.658 0.031	9.281 0.002	3.717 0.054
Anxiety	13.928 0.000	11.254 0.001	2.267 0.132	14.898 0.000	4.619 0.032
Stress	5.506 0.019	7.490 0.006	0.613 0.434	1.138 0.286	3.766 0.052

The first value is the χ2 statistic; the second value is the significance (r).

**Table 8:** Analysis of the most influential variable on eating disorders.

	Cox & Snell R Square	Nagelkerke R Square	Predictor Variable	Wald	*p*	Exp(B)	Lower 95% CI	Upper 95% CI
All respondents (N = 189)	0.073	0.203	Depressiveness	2.285	0.131	1.146	0.960	1.368
Girls (N = 104)	0.151	0.361	Weight	6.096	0.014	0.895	0.820	0.977
Boys (N = 85)	0.069	0.263	Depressiveness	3.286	0.070	1.417	0.972	2.067
Primary school students (N = 69)	0.224	0.744	Anxiety	1.289	0.256	8.941	0.204	392.2
Secondary school students (N = 120)	0.072	0.185	Depressiveness	1.739	0.187	1.132	0.941	1.362

Exp(B)—Odds ratio; CI—Confidence interval.

## Data Availability

The original contributions presented in this study are included in the article. Further inquiries can be directed to the corresponding author.
